# Mathematical modeling of the performance of wild and transgenic mosquitoes in malaria transmission

**DOI:** 10.1371/journal.pone.0285000

**Published:** 2023-04-28

**Authors:** Ana Paula Wyse, Antonio José Boness dos Santos, Juarez dos Santos Azevedo, Aline Costa de Meneses, Victor Matheus Da Cunha Santos

**Affiliations:** 1 Department of Scientific Computing, Informatics Center, Federal University of Paraíba, João Pessoa, Paraíba, Brazil; 2 Institute of Science, Technology and Innovation, Federal University of Bahia, Camaçari, Bahia, Brazil; 3 Graduate Program in Mathematical and Computational Modeling, Informatics Center, Federal University of Paraíba, João Pessoa, Paraíba, Brazil; University of Cape Coast, GHANA

## Abstract

A mathematical model that simulates malaria transmission under the influence of transgenic mosquitoes refractory to malaria is presented in this paper. The zygosity of transgenic mosquitoes is taken into account and, consequently, the total population of mosquitoes is comprised of wild type and heterozygous and homozygous transgenic mosquitoes. These three mosquito varieties interact by mating and competition, and the genetic characteristics of their resulting offspring are in accordance with Mendelian genetics or the mutagenic chain reaction (MCR) technique. Although the incorporation of transgenic mosquitoes into the ecosystem reduces the incidence of malaria, the model also takes into account the importance of completing treatment in individuals with confirmed infection and the imminent risk of increased environmental temperature.

## 1. Introduction

Considerable efforts have been expended to improve the methods used to combat tropical diseases, particularly those transmitted by vectors comprising malaria, dengue, Chicungunya and Zika virus. Due to the alarming number of cases reported annually in Africa, the Amazon and some regions of Southeast Asia, researchers have sought over the years to better understand the factors linked to the transmission of these diseases and so intervene in various ways to block the contagion. The fight against mosquito-borne diseases encompass a set of actions to be implement [1]. Over time, isolated measures can become ineffective.

Well-known measures, such as the use of insecticide sprayers and medicines, are becoming obsolete as mosquitoes are becoming resistant to insecticides [2], and parasites have evolved in such a way that they are not detected and/or fail to complete the endogenous cycle, evading the human immune system [3]. According to the World Health Organization, mosquito resistance to insecticides is a major concern, particularly for pyrethroids, which are used on bed nets [4]. The evolution of the protozoan is notorious for the prolongation of its infectious period in patients who respond unfavorably to medication due to drug resistance. *Plasmodium falciparum* resistance to chloroquine and artemisinins has been found (see Ref. [5]) and, although chloroquine is still used to treat malaria induced by *Plasmodium vivax*, *P. vivax* drug resistance has also been reported, leading some countries to adopt artemisinin-based combination therapies (ACTs) to medicate malaria patients. In addition, studies about the *P. falciparum* parasite have revealed that a large proportion of this protozoan lacks the pfhrp2 gene [6]. Without this gene, *P. falciparum* cannot be detected by HRP2-based rapid diagnostic tests. Since 2016, the WHO database has included pfhrp2/3 gene deletion data for 20 countries.

Due to the progress made in recent years in genetic manipulation, the use of transgenic mosquitoes may be sucessfull option for composing a set of viable disease-fighting actions. Arthropod control by genetic manipulation is an old idea that arose in the 1930s-1940s by 3 pioneers independently researchings: A. S. Serebrovskii [7], F. L. Vanderplank [8], and E. F. Knipling [9], and studies on sterile males were highlighted during the 1970s (see Ref. [10], for example), which encouraged re-evaluation of the original ideas. A review of vector control by genetic manipulation can be found in Refs. [11–13].

Substantial progress has since been made to increase the vector population frequency of genes that interfere with pathogen development [14]. The first malaria-refractory *Anopheles* mosquitoes were designed in 2002 using an innovative technique developed by Catteruccia et al. [15]. Two different transgenic *Anopheles stephensi* lines were developed using the CP (carboxypeptidase) promoter: the first line expressed the synthetic peptide SM1 (salivary gland and midgut binding peptide 1) [16], and the second line expressed an enzyme present in bee venom PLA2 (phospholipase A2) [17]. On the one hand, according to Moreira et al. [18], the life span, fecundity and fertility of females expressing the SM1 tetramer exhibit no significant fitness load when compared with their nontransgenic relatives. On the other hand, mosquitoes expressing the PLA2 transgene had a significant reduction of fecundity due to insufficient blood ingestion. This technique was used in 2010 by Amenya et al. [19] to get *A. stephensi* transgenic lineages carrying *ϕ*C31 attP “docking” sites associated to a fluorescent marker gene. In 2014, Vega-Rodríguez et al. [20] identified a mosquito midgut receptor called enolase-binding protein (EBP) which associates both SM1 and ookinete surface enolase, proving the efficiency of SM1.

Successful transgene fixation conditions can be obtained by the mutagenic chain reaction (MCR) technique proposed by Gantz et al. [21]. The goal of this technique is to suppress wild populations by converting heterozygous organisms into homozygous transgenic organisms before they reach adulthood. The efficiency of MCR inheritance was proven using *Drosophila melanogaster* [21] and then for *A. stephensi*, verifying higher drive efficiency for lines resulting from the crossing of drive-males with wild-females [22, 23].

Mathematical models describing the interaction between wild and genetically modified mosquitoes, as well as their spread, have been widely studied in the literature (see for example [24] and references therein). Recently, Wyse et al. [25] presented a stochastic reaction-diffusion model with resistant allele dynamics based on the MCR technique. In this paper, these dynamics are linked to an susceptible-exposed-infectious(SEI) compartmental model for mosquitoes and a susceptible-exposed-infectious-susceptible (SEIS) model for human recovery based on treatment completion. The resulting mathematical model is used to investigate the potential effects of transgenic mosquitoes refractory to malaria on disease transmission while maintaining conventional drug treatment.

## 2. Materials and methods

This research was carried out exclusively with scientific texts, and therefore no approval by an ethics committee was required, according to Resolution 510/2016 (article 1, section VI) from the National Health Council (CNS), Brazil.

The proposed mathematical model consists of SEIS dynamics describing the infectious process in humans and an SEI model describing the infectious process in mosquitoes. Although acquired immunity is relevant, there is no simple relationship between parasitaemia and the individual’s previous exposure to the disease, for this reason the model considers no immunity, representing a worse epidemiological situation. Both human and mosquito are represented by compartments subdivided into classes that interact, prompting flux between them, starting with the initiation of the infectious dynamic. The adopted strategy is to engineer fertile transgenic mosquitoes refractory to malaria and able to transmit the gene that blocks the protozoan through mating with wild mosquitoes.

### 2.1 Governing equations for mosquitoes dynamics

An interaction model of wild and transgenic mosquitoes was previously proposed by Wyse et al. [25]. Let populations *U*_*i*_ (*i* = 1, 2, 3) representing the populations of wild, heterozygous and homozygous transgenic mosquitoes. Assuming these three populations have equal fitness, each mosquito (wild or transgenic) reaches adulthood at a certain rate *ϵ* and exits its population owing to density-dependent or density-independent death. Density-dependent death ocurs when there is competition for resource, and its rate is $\frac{r\sum U_{i}}{k}$, where *r* is the intrinsic increase rate and *k* is the carrying capacity of the mosquitoes environment. These dynamics occur for each subpopulation *U*_*i*_, over time.

Since mosquitoes are diploid organisms, we can establish (*w*, *w*) for the wild mosquito genotype, (*g*, *g*) for the homozygous transgenic mosquito genotype and (*g*, *w*) for the heterozygous transgenic mosquito genotype. For *i*, *j* = 1, 2, 3, mating *U*_*i*_ × *U*_*j*_ can generate populations *U*_1_, *U*_2_ and *U*_3_ with genotypic frequencies *a*_*ij*_, *b*_*ij*_ and *c*_*ij*_, respectively. Table 1 displays the genotypic offspring frequencies resulting from all mating combinations among the different *U*_*i*_.

**Table 1:** Genotypic offspring frequencies obtained from mating wild, heterozygous and homozygous transgenic mosquitoes.

Genotype	*U*_1_ × *U*_1_	*U*_1_ × *U*_2_	*U*_1_ × *U*_3_	*U*_2_ × *U*_2_	*U*_2_ × *U*_3_	*U*_3_ × *U*_3_
Mating						
(*w*, *w*)	*a*_11_	*a*_12_	*a*_13_	*a*_22_	*a*_23_	*a*_33_
(*w*, *g*)	*b*_11_	*b*_12_	*b*_13_	*b*_22_	*b*_23_	*b*_33_
(*g*, *g*)	*c*_11_	*c*_12_	*c*_13_	*c*_22_	*c*_23_	*c*_33_

From the assumptions above, the differential ordinary equations governing the behavior dynamics of the three mosquitoes varieties is represented by the system:

$$\begin{array}{c} \left\{ \begin{array}{cc} \frac{dU_{1}}{dt} & =(\frac{\epsilon }{\sum U_{i}}-\frac{r}{k})\sum \sum a_{ij}U_{i}U_{j}-\delta U_{1},\\ \frac{dU_{2}}{dt} & =(\frac{\epsilon }{\sum U_{i}}-\frac{r}{k})\sum \sum b_{ij}U_{i}U_{j}-\delta U_{2},\\ \frac{dU_{3}}{dt} & =(\frac{\epsilon }{\sum U_{i}}-\frac{r}{k})\sum \sum c_{ij}U_{i}U_{j}-\delta U_{3}, \end{array}\right. \end{array}$$ (1)

with initial conditions *U*_*i*_(0) = *U*_*i*0_. Additional details about this modeling of behavior dynamics can be found in Ref. [25].

Summing the three equations (1), we obtain the logistic equation with harvesting to describe the total mosquito population

$$\begin{array}{c} \frac{dU}{dt}=rU(1-\frac{U}{k})-\delta_{2}U, \end{array}$$ (2)

where *U* = ∑*U*_*i*_ and *r* = *ε* − *δ*_1_, with *ε* being the rate at which mosquitoes enter adulthood and *δ*_1_ being the death rate from natural causes. The density-independent death rate, *δ*_2_, acts as a harvest rate, and ensures that the population is stabilized bellow carrying capacity *k*, at non-zero equilibrium point

$$\begin{array}{c} U^{*}=k(1-\frac{\delta_{2}}{r}). \end{array}$$ (3)

The coefficients of Eq (2) that describes the population dynamics of mosquitoes were adjusted to represent the field situation [26, 27], presenting accurate results. The mathematical models used in some studies for malaria transmission suggesting a logistic form of intraspecific competition to describe the variable mosquito populations(see Refs. [27–29] for examples).

### 2.2 Modeling the malaria transmission under the influence of transgenic mosquitoes

The essential element for the transmission of malaria is the bite of female *Anopheles* mosquitoes. Thus, the interaction between humans and mosquitoes is a determining factor in assessing the risk of contagion of the disease. When a human is infectious, their blood contains sexual forms of the pathogen, called gametocytes, which are ingested by the mosquito during the bite and, after a few days, these forms evolve until they reach the sporozoite stage and migrate to the salivary glands of the mosquito, making it infectious. Transmission is completed when the infectious mosquito bites a susceptible human, injecting saliva contaminated with *Plasmodium* sporozoites into his bloodstream. After a few days, the sporozoites evolve to the gametocyte stage and begin to circulate in the bloodstream, making the human infectious. However, if neither the mosquito nor the human being is infected by the malarial protozoan, there is no transmission. For more details see [30].

To describe the interaction between humans and mosquitoes, the mathematical model represented by the system in (1) is linked with the mathematical model for malaria transmission proposed by Wyse et al., [27] using different treatment intensities but a nonseasonal environment. The combined model works under the following assumptions:

- The human population *H* consists of susceptible (*H*_*S*_), exposed (*H*_*E*_) and infectious (*H*_*I*_) individuals;
- The mosquito population is composed of wild (*U*_1_) and transgenic (heterozygous–*U*_2_ and homozygous–*U*_3_) individuals;
- Each subpopulation of mosquitoes, *U*_*i*_, is composed of susceptible (*U*_*iS*_), exposed (*U*_*iE*_) and infectious (*U*_*iI*_) individuals;
- The contact between infectious mosquitoes *U*_*iI*_ and susceptible humans induces a disease transmission, which will be well succeeded as this contact is effective;
- Transgenic mosquitoes have a reduced degree of infectiousness compared to wild mosquitoes;
- The dominance of the *g* or *w* allele defines whether the phenotype of heterozygous mosquitoes is to be refractory to malaria or not.

Starting from these premises, the human population is described by the equations

$$\left\{\begin{array}{l} \frac{dH_{S}}{dt}=\mu H-\sum \alpha \beta U_{iI}\frac{H_{S}}{H}-\mu H_{S}+\overset{n}{\underset{\kappa =1}{\sum }}\left(\phi_{\kappa }p_{\kappa }\right)H_{I}\\ \frac{dH_{E}}{dt}=\sum \alpha \beta U_{iI}\frac{H_{s}}{H}-\mu H_{E}-\eta H_{E}\\ \frac{dH_{I}}{dt}=\eta H_{E}-\mu H_{I}-\overset{n}{\underset{\kappa =1}{\sum }}\left(\phi_{\kappa }p_{\kappa }\right)H_{I} \end{array}\right.$$ (4)

Here, the human population size is constant so that no migration is considered and *μ* is both human birth and death rate per time unit. The coefficient *α* is the average biting rate of each mosquito per time unit while the probability of infection *β* ∈ [0, 1] represents the proportion of infectious bites on susceptible humans that effectively produce infection. For each variety *i* there are *U*_*i*_*H* bites per human per unit of time. Assuming that there are *H*_*S*_ susceptible humans and that the proportion of the total number of bites that are potentially infectious to humans is *U*_*iI*_*U*_*i*_, the number of potentially infectious bites given in susceptible humans is *αU*_*iI*_*H*_*S*_*H* bites per unit time. However, only a *β* fraction of these bites actually infect humans.

The expected value of the intrinsic latent period is 1/*η*, where *η* is the infectious rate and the proportion of infected humans undergoing treatment *κ* is *p*_*κ*_ ∈ [0, 1], where $\sum_{\kappa =1}^{n}p_{\kappa }=1$. The expected value of the infectious period for *p*_*κ*_ is 1/*ϕ*_*κ*_, where *ϕ*_*κ*_ is the disease recovery rate of infected people undergoing *κ*-treatment.

From (1), the equation relative to wild mosquitoes, *U*_1_, can be rewritten as

$$\begin{array}{c} \frac{dU_{1}}{dt}=\varepsilon \frac{\sum \sum a_{ij}U_{i}U_{j}}{\sum U_{i}}-\frac{r}{k}\frac{\sum \sum a_{ij}U_{i}U_{j}}{U_{1}}U_{1}-\delta U_{1}. \end{array}$$ (5)

The first factor, $\varepsilon \frac{\sum \sum a_{ij}U_{i}U_{j}}{\sum U_{i}}$ refers to the recruitment rate of wild mosquitoes to the adult stage; the second factor, $\frac{r}{k}\frac{\sum \sum a_{ij}U_{i}U_{j}}{U_{1}}U_{1}$, refers to density-dependent mortality caused by limited resources; the third factor, *δU*_1_, refers to density-independent mortality. As all individuals are born susceptible, the first factor belongs fully to the equation of susceptible wild mosquitoes. Assuming that the competition for resources affects all mosquitoes equally, the second factor should be proportionally divided by the three subpopulations of wild mosquitoes *U*_1*S*_, *U*_1*E*_ and *U*_1*I*_ as

$$\left\{\begin{array}{l} \frac{dU_{1S}}{dt}=\varepsilon \frac{\sum \sum a_{ij}U_{i}U_{j}}{\sum U_{i}}-\frac{r}{k}\frac{\sum \sum a_{ij}U_{i}U_{j}}{U_{1}}U_{1S}-\delta U_{1S}-\alpha \zeta_{1}U_{1S}\frac{H_{I}}{H}\\ \frac{dU_{1E}}{dt}=\alpha \zeta_{1}U_{1S}\frac{H_{I}}{H}-\frac{r}{k}\frac{\sum \sum a_{ij}U_{i}U_{j}}{U_{1}}U_{1E}-\delta U_{1E}-\gamma \left(T\right)U_{1E}\\ \frac{dU_{1I}}{dt}=\gamma \left(T\right)U_{1E}-\frac{r}{k}\frac{\sum \sum a_{ij}U_{i}U_{j}}{U_{1}}U_{1I}-\delta U_{1I} \end{array}\right.$$ (6)

The probability of infection *ζ*_*i*_ is the proportion of bites of uninfected mosquitoes on infectious humans that produce effectivelly an infection for a mosquito of variety *i*. The duration of the extrinsic latent period is 1/*γ*(*T*), where *γ*(*T*) is the rate at which exposed mosquitoes become infectious per time unit and *T* is the environmental temperature.

Using analogous reasoning, we obtain equations that describe the dynamics of the heterozygous and homozygous transgenic mosquitoes given by (7) and (8), respectively.

$$\left\{\begin{array}{l} \frac{dU_{2S}}{dt}=\varepsilon \frac{\sum \sum b_{ij}U_{i}U_{j}}{\sum U_{i}}-\frac{r}{k}\frac{\sum \sum b_{ij}U_{i}U_{j}}{U_{2}}U_{2S}-\delta U_{2S}-\alpha \zeta_{2}U_{2S}\frac{H_{I}}{H}\\ \frac{dU_{2E}}{dt}=\alpha \zeta_{2}U_{2S}\frac{H_{I}}{H}-\frac{r}{k}\frac{\sum \sum b_{ij}U_{i}U_{j}}{U_{2}}U_{2E}-\delta U_{2E}-\gamma \left(T\right)U_{2E}\\ \frac{dU_{2I}}{dt}=\gamma \left(T\right)U_{2E}-\frac{r}{k}\frac{\sum \sum b_{ij}U_{i}U_{j}}{U_{2}}U_{2I}-\delta U_{2I} \end{array}\right.$$ (7)

$$\left\{\begin{array}{l} \frac{dU_{3S}}{dt}=\varepsilon \frac{\sum \sum c_{ij}U_{i}U_{j}}{\sum U_{i}}-\frac{r}{k}\frac{\sum \sum c_{ij}U_{i}U_{j}}{U_{3}}U_{3S}-\delta U_{3S}-\alpha \zeta_{3}U_{3S}\frac{H_{I}}{H}\\ \frac{dU_{3E}}{dt}=\alpha \zeta_{3}U_{3S}\frac{H_{I}}{H}-\frac{r}{k}\frac{\sum \sum c_{ij}U_{i}U_{j}}{U_{3}}U_{3E}-\delta U_{3E}-\gamma \left(T\right)U_{3E}\\ \frac{dU_{3I}}{dt}=\gamma \left(T\right)U_{3E}-\frac{r}{k}\frac{\sum \sum c_{ij}U_{i}U_{j}}{U_{3}}U_{3I}-\delta U_{3I} \end{array}\right.$$ (8)

Mosquito population modification involves the introduction of genes that give the species a phenotype of resistance to the malarial protozoan. Thus, mosquitoes of the *U*_3_ variety were conceived to present such resistance when compared to the wild equivalent *U*_1_; this resistance is represented in the coefficients *ζ*_*i*_ in the Eqs 6–8, where the inequality *ζ*_3_ < *ζ*_1_ must be satisfied. The lower the *ζ*_*i*_ coefficient, the lower the malaria transmission rate. The phenotype of mosquitoes of the heterozygous variety *U*_2_ could be the same as *U*_1_ if the *w* allele is dominant, the same as *U*_3_ if the *g* allele is recessive or intermediate and, in the latter case, *ζ*_3_ < *ζ*_2_ < *ζ*_1_.

## 3 Model analysis

### 3.1 The basic reproductive rate

To qualitatively analyze the dynamics of malaria, the system (6)–(8) can be made dimensionless by defining new variables as follows:

$$\begin{array}{c} h_{S}=\frac{H_{S}}{H};\;h_{E}=\frac{H_{E}}{H};\;h_{I}=\frac{H_{I}}{H};\;u_{iS}=\frac{U_{iS}}{U};\;u_{iE}=\frac{U_{iE}}{U};\;u_{iI}=\frac{U_{iI}}{U}. \end{array}$$ (9)

Clearly, with these new variables, *h*_*S*_ + *h*_*E*_ + *h*_*I*_ = 1 and $\sum_{i=1}^{3}\left(u_{iS}+u_{iE}+u_{iI}\right)=1$. Thus, substituting the new variables (9) into the system (6)–(8) and taking into account that the total mosquito population quickly stabilizes at the equilibrium state (3), we have

$$\left\{\begin{array}{l} \frac{dh_{S}}{dt}=\mu -\sum \alpha \beta u_{iI}h_{S}m-\mu h_{S}+\overset{n}{\underset{\kappa =1}{\sum }}\left(\phi_{\kappa }p_{\kappa }\right)h_{I}\\ \frac{dh_{E}}{dt}=\sum \alpha \beta u_{iI}h_{S}m-\mu h_{E}-\eta h_{E}\\ \frac{dh_{I}}{dt}=\eta h_{E}-\mu h_{I}-\overset{n}{\underset{\kappa =1}{\sum }}\left(\phi_{\kappa }p_{\kappa }\right)h_{I} \end{array}\right.$$ (10)

$$\left\{\begin{array}{l} \frac{du_{iS}}{dt}=\left(\varepsilon -\left(\varepsilon -\delta \right)\frac{u_{iS}}{u_{i}}\right)\left(\sum \sum ϒu_{i}u_{j}\right)-\delta u_{iS}-\alpha \zeta_{i}u_{iS}h_{I}\\ \frac{du_{iE}}{dt}=\alpha \zeta_{i}u_{iS}h_{I}-\left(\varepsilon -\delta \right)\frac{u_{iE}}{u_{i}}\left(\sum \sum ϒu_{i}u_{j}\right)-\delta u_{iE}-\gamma \left(T\right)u_{iE}\\ \frac{du_{iI}}{dt}=\gamma \left(T\right)u_{iE}-\left(\varepsilon -\delta \right)\frac{u_{iI}}{u_{i}}\left(\sum \sum ϒu_{i}u_{j}\right)-\delta u_{iI} \end{array}\right.$$ (11)

where *h*_*S*_, *h*_*E*_, *h*_*I*_ are the fractions of susceptible, exposed and infectious humans in relation to the total human population, respectively, and *u*_*i*_ (*i* = 1, 2, 3) are the fractions relative to the total mosquito population of each one of the three mosquitoes varieties considered in the model, in accordance with its zigozity (wild, heterozygous and homozygous transgenic mosquitoes), where *u*_*i*_ = *u*_*iS*_ + *u*_*iE*_ + *u*_*iI*_. The symbol *Υ* must be substituted by *a*_*ij*_, *b*_*ij*_ or *c*_*ij*_ depending on whether the set of equations is modeling wild (*u*_1_), heterozygous (*u*_2_) or homozygous transgenic mosquitoes (*u*_3_). The ratio of female mosquitoes per human host is $m=\frac{U}{H}$.

Disease evolution trends in a susceptible population can be assessed by the base reproductive number, *R*_0_. This is an important parameter that indicates how the disease progresses over time. There are three possible paths: if *R*_0_ = 1 the disease will stabilize at an endemic level, if *R*_0_ < 1 the complete eradication will be attained, if *R*_0_ > 1 the entire population will be contamined.

With respect to diseases transmitted by microparasites, *R*_0_ is the estimated average amount of secondary infections produced from a single infected individual inserted into a fully susceptible population (humans and mosquitoes), during its period and infectivity.

If any host population involved in the process consists of both susceptible and nonsusceptible hosts, the effective reproductive number, *R*, estimates the average amount of secondary infections produced by an infective agent, $R=R_{0}\prod_{\rho =1}^{m}n_{\rho }$, where *n*_*ρ*_ is the fraction of susceptible individuals in the host population *ρ*. If all the individuals in population *ρ* are susceptible to the disease, then *n*_*ρ*_ = 1 and, if this holds for all the populations involved in transmission *R* = *R*_0_. For the mathematical model proposed here involving two host populations, we have *R* = *R*_0_*n*_1_*n*_2_, where *n*_1_ and *n*_2_ are fractions of the susceptible host population from species 1 (humans) and 2 (mosquitoes) at steady state.

The basic reproductive number is usually derived algebraically by the stability properties of the system, being mathematical defined as the dominant eigenvalue of a positive linear operator [31]. An alternative derivation can be obtained by analysing the phase—plane of the dynamical behavior of the model [30].

Here, we obtain an expression for the basic reproductive rate by analyzing isoclines of infectious humans and mosquitoes. The equilibrium state is represented by the intersection of the two isoclines, which is a converging point $\left(h_{I}^{*},u_{1I}^{*}\right)$ of a subset of trajectories.

To facilitate drawing the isoclines, each particular pair of values (*h*_*I*_, *u*_1*I*_) is corresponding to a point of a standard two-dimensional graph where the horizontal axis refers to the variable *h*_*I*_(*t*) and the vertical axis refers to the variable *u*_1*I*_.

Requiring that $\frac{dh_{S}}{dt}=\frac{dh_{E}}{dt}=\frac{dh_{I}}{dt}=0$ and solving the system for *h*_*S*_, *h*_*E*_, *u*_1*I*_, the *h*_*I*_ isocline is given by

$$\begin{array}{c} u_{1I}^{*}\left(t\right)=-\frac{h_{I}^{*}(\mu^{2}+(\mu +\eta )\sum_{\kappa =1}^{n}\left(\phi_{\kappa }p_{\kappa }\right)+\mu \eta )}{\alpha \beta m(-\eta +(\mu +\eta +\sum_{\kappa =1}^{n}\left(\phi_{\kappa }p_{\kappa }\right))h_{I}^{*})}. \end{array}$$ (12)

In addition, requiring that $\frac{du_{1S}}{dt}=\frac{du_{1E}}{dt}=\frac{du_{1I}}{dt}=0$ and solving the system for *u*_1*S*_, *u*_1*E*_, and *u*_1*I*_, the *u*_1*I*_ isocline is given by:

$$\begin{array}{c} u_{1I}^{*}\left(t\right)=\frac{\alpha \zeta_{1}h_{I}^{*}\gamma }{\alpha \zeta_{1}h_{I}^{*}\gamma +\alpha \zeta_{1}h_{I}^{*}\varepsilon +\varepsilon \gamma +\varepsilon^{2}}, \end{array}$$ (13)

In this way, the disease-free equilibrium, given by $P_{0}=\left(h_{S}^{*},0,0,u_{1S}^{*},0,0\right)$, and the endemic equilibrium, given by $P_{1}=\left(h_{S}^{*},h_{E}^{*},h_{I}^{*},u_{1S}^{*},u_{1E}^{*},u_{1I}^{*}\right)$ are identified.

When *h*_*I*_ isoclines and *u*_1*I*_ isocline intersect, this point represents the steady state of the process. This intersection will occur at positive values of *h*_*I*_ and *u*_1*I*_ whenever the initial slope of the *u*_1*I*_ isocline exceeds that of the *h*_*I*_ isocline. Based on the second-order derivative, we can confirm that the graph of the *h*_*I*_ isocline is concave up, and the concavity of the *u*_1*I*_ isocline is down.

At *h*_*I*_ = 0, the slope of the *h*_*I*_ isocline is

$$\begin{array}{c} s_{h}=\frac{\left(\mu^{2}+(\mu +\eta )\sum_{\kappa =1}^{n}\left(\phi_{\kappa }p_{\kappa }\right)+\eta \mu \right)}{\alpha \beta m\eta }, \end{array}$$ (14)

and the slope of the *u*_1*I*_ isocline is

$$\begin{array}{c} s_{u}=\frac{\alpha \zeta_{1}\gamma }{(\gamma +\varepsilon )\varepsilon }. \end{array}$$ (15)

**Fig 1:**
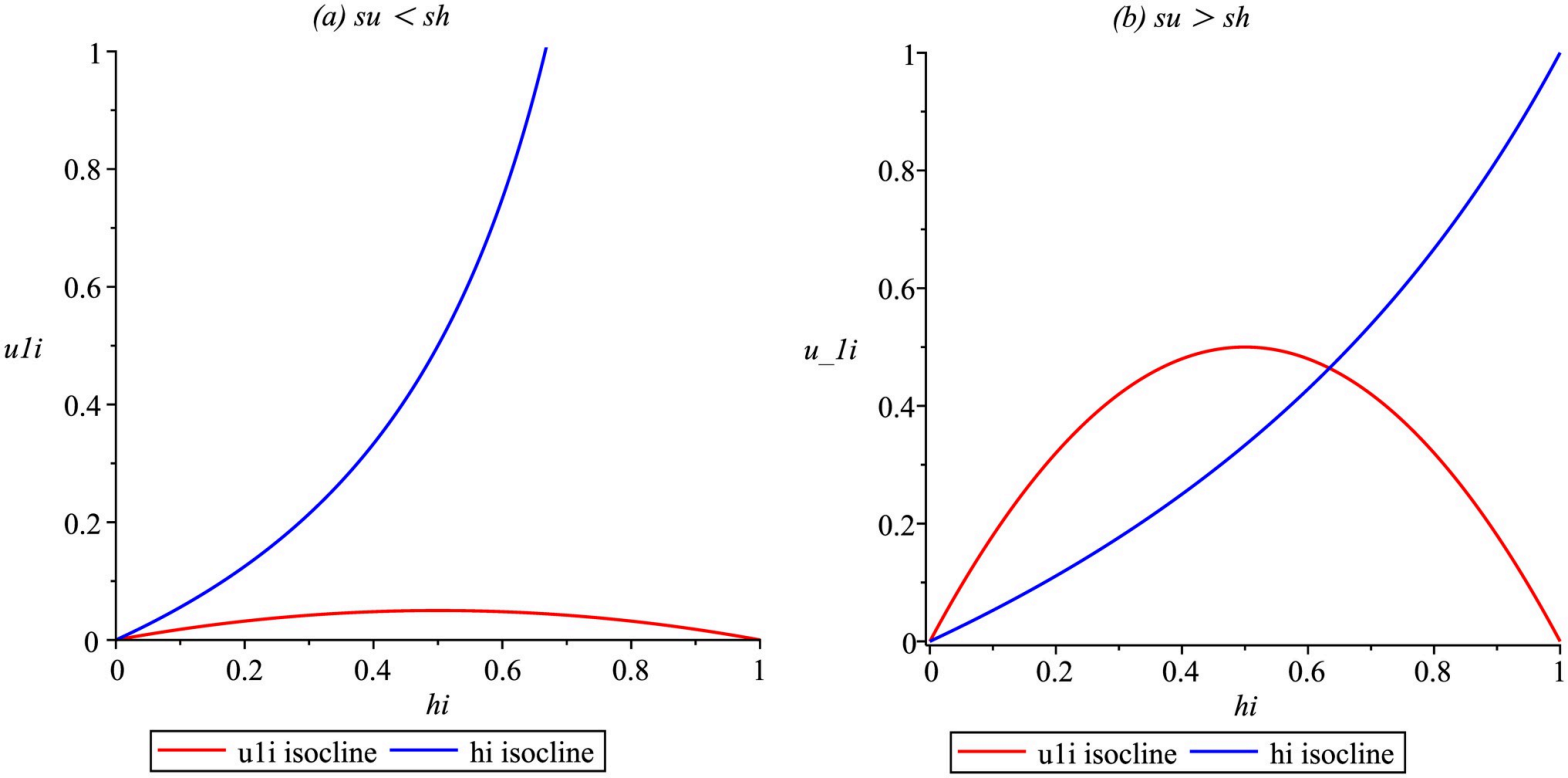
Illustration of the possible behaviors of the isoclines u_1I_ and h_I_. Initial slope of *u*_1*I*_ below the slope of *h*_*I*_ isocline (a) and initial slope of *u*_1*I*_ above the slope of *h*_*I*_ isocline (b).

When *s*_*u*_ < *s*_*h*_, the initial slope of the *u*_1*I*_ isocline lies below the slope of the *h*_*I*_ isocline (see Fig 1a for ilustration); then *R*_0_ < 1 and the infection cannot persist. On the other hand, *s*_*u*_ > *s*_*h*_ meand that the initial slope of the *u*_1*I*_ isocline lies above the slope of the *h*_*I*_ isocline (see Fig 1b for ilustration), then *R*_0_ > 1 and the disease is endemic.

For *s*_*u*_ > *s*_*h*_, the inequality

$$\begin{array}{c} \frac{\frac{\alpha \zeta_{1}\gamma }{(\gamma +\varepsilon )\varepsilon }}{(\mu^{2}+(\mu +\eta )\sum_{\kappa =1}^{n}\left(\phi_{\kappa }p_{\kappa }\right)+\eta \mu ){\left(\alpha \beta m\eta \right)}^{-1}}>1 \end{array}$$ (16)

is satisfied and

$$\begin{array}{c} R_{0}=\frac{\gamma \alpha^{2}\zeta_{1}\beta m\eta }{\varepsilon \left(\gamma +\varepsilon \right)(\mu^{2}+(\mu +\eta )\sum_{\kappa =1}^{n}\left(\phi_{\kappa }p_{\kappa }\right)+\eta \mu )} \end{array}$$ (17)

is the mathematical expression for basic reproductive number when no transgenic mosquitoes is in the process.

The presence of transgenic mosquitoes implies that a proportion of the mosquito population is not susceptible to malaria. In this situation, considering no immunity is conferred to humans, the effective reproductive number is

$$\begin{array}{c} R=\frac{\gamma \alpha^{2}\zeta_{1}\beta m\eta }{\varepsilon \left(\gamma +\varepsilon \right)(\mu^{2}+(\mu +\eta )\sum_{\kappa =1}^{n}\left(\phi_{\kappa }p_{\kappa }\right)+\eta \mu )}n_{2}=R_{0}\left(u_{1S}^{*}+u_{2S}^{*}+u_{3S}^{*}\right). \end{array}$$ (18)

The relationship $R=R_{0}\sum_{i=1}^{3}u_{iS}^{*}$ given by Eq (18) signifies the introduction of transgenic mosquitoes into the system to reduce disease incidence acts by reducing the basic reproductive number so that *u*_*iS*_ ∈ [0, 1], and we have *R* ≤ *R*_0_.

An heuristic derivation of *R* considering transgenic mosquitoes is as follows. If we are considering Mendelian genetics, the equilibrium state of wild and transgenic mosquitoes is at Hardy–Weinberg equilibrium (*r*^2^, 2*rq*, *q*^2^) where *r* and *q* are the initial allele frequencies of *w* and *g*, respectively. Therefore, for initial conditions (*u*_10_, *u*_20_, *u*_30_), the equilibrium state is $\left(u_{1}^{*},u_{2}^{*},u_{3}^{*}\right)=\left({\left(u_{10}+\frac{u_{20}}{2}\right)}^{2},2\left(u_{10}+\frac{u_{20}}{2}\right)\left(u_{30}+\frac{u_{20}}{2}\right),{\left(u_{30}+\frac{u_{20}}{2}\right)}^{2}\right)$. Thus, for classical Mendelian genetics, the effective reproductive number is

$$\begin{array}{c} R_{Mend}=R_{0}\left({\left(u_{10}+\frac{u_{20}}{2}\right)}^{2}\left(1-\tau_{1}\right)+2\left(u_{10}+\frac{u_{20}}{2}\right)\left(u_{30}+\frac{u_{20}}{2}\right)\left(1-\tau_{2}\right)+{\left(u_{30}+\frac{u_{20}}{2}\right)}^{2}\left(1-\tau_{3}\right)\right), \end{array}$$ (19)

where *τ*_*i*_ ∈ [0, 1] depends on the degree of malaria refractoriness conferred on each mosquito variety. In particular, *τ*_1_ = 0, meaning that the wild population is completely susceptible to malaria.

For the MCR technique, there are three possibilities:

1.
$\left(u_{1}^{*},u_{2}^{*},u_{3}^{*}\right)=\left(0,0,1\right)$ is a fixed point and *R*_*MCR*_ = *R*_0_(1 − *τ*_3_);
2.
$\left(u_{1}^{*},u_{2}^{*},u_{3}^{*}\right)=\left(1,0,0\right)$ is a fixed point and *R*_*MCR*_ = *R*_0_;
3.
$\left(u_{1}^{*},u_{2}^{*},u_{3}^{*}\right)$ is a fixed point that indicates coexistence between the 3 varieties of mosquitoes and $R_{MCR}=R_{0}\left(u_{1}^{*}+u_{2}^{*}\left(1-\tau_{2}\right)+u_{3}^{*}\left(1-\tau_{3}\right)\right)$.

Let *x* represent the proportion of mosquitoes that are nonsusceptible to malaria. Then, $\sum_{i=1}^{3}u_{iS}^{*}=1-x$, implying that *R*_0_(1 − *x*) < 1 must be satisfied to eliminate disease. From this relation, find that the herd immunity threshold is $x>1-\frac{1}{R_{0}}$. This threshold is an important measure guiding the genetic modification needed to obtain at least *x* percent of transgenic mosquitoes refractory to malaria disease reduction.

### 3.2 Sensitivity analysis of *R*_0_

Local sensitivity analysis of the basic reproductive number allows us to determine the relative importance of the different epidemiological factors involved. As *R*_0_ is differentiable in relation to each of its parameters, we can carry out local sensitivity analysis using the simple approach

$$\begin{array}{c} \Omega_{\theta }^{R_{0}}=\frac{\partial R_{0}}{\partial \theta }\times \frac{\theta }{R_{0}}, \end{array}$$ (20)

where *R*_0_ is defined in (17), and *θ* represents each of the model parameters.

Using the parameter values presented in Table 2, we can compute local sensitivity indices using Eq (20) and show the impact of each parameter on the reproductive number in Table 3.

**Table 2:** Model coefficients obtained directly or estimated from Wyse et al. [27] and Detinova et al. [32].

Coefficients	Values
*ε*	8.3484/month
*δ*_1_	4/month
*δ*_2_	2.545/month
*μ*	0.0014
*α*	5.974/month
*β*,*ζ*_1_	0.3
*η*	3/month
*ϕ*_1_	1.5
*ϕ*_2_	0.0832
*ϕ*_3_	0
*γ*	$\frac{T-14.5}{3.5}$ /month
*m*	11.57
*k*	279000

**Table 3:** Sensitivity indices of R_0_ to the malaria model parameters evaluated at the baseline parameter values given in Table 2.

Parameters: *θ*	Sensitivity index(SI): $\Omega_{\theta }^{R_{0}}$	SI evaluated on parameters
*T*	$\frac{0.286\varepsilon T}{\left(0.286T-4.143)(0.286T-4.143+\varepsilon \right)}$	$\frac{29.22T}{\left(T-14.5)(T+14.72\right)}$
*α*	2	2
*β*,*ζ*_1_, *m*	1	1
*η*	$\frac{\mu }{\mu +\eta }$	0.00046
*ε*	$-\frac{0.286T-4.143+2\varepsilon }{0.286T-4.143+\varepsilon }$	$-\frac{0.286T+12.554}{0.286T+4.205}$
*μ*	$-\frac{(2\mu +\sum_{\kappa =1}^{n}\phi_{\kappa }p_{\kappa }+\eta )\mu }{\mu^{2}+\mu \sum_{\kappa =1}^{n}\phi_{\kappa }p_{\kappa }+\eta \mu +\eta \sum_{\kappa =1}^{n}\phi_{\kappa }p_{\kappa }}$	$-\frac{0.0014(3.0028+1.5p_{1}+0.0832p_{2})}{0.0042+4.5021p_{1}+0.2497p_{2}}$
*ϕ*_*κ*_, *p*_*κ*_	$-\frac{\phi_{\kappa }p_{\kappa }}{\mu +\sum_{\kappa =1}^{n}\phi_{\kappa }p_{\kappa }}$	$-\frac{\phi_{\kappa }p_{\kappa }}{0.0014+1.5p_{1}+0.0832p_{2}}$

The biting rate, *α*, one of the most important coefficients in the model ($\Omega_{\alpha }^{R_{0}}=2$) has a high impact on model outcomes independent of assigned parameter values. Increasing or decreasing *α* by 10%, for example, can increase or decrease *R*_0_ by 20%. In contrast, *R*_0_ is virtually insensitive to variations in *η*.

Some sensitivity indices may vary according to temperature or the proportion of people cured due to a specific set of actions. Such cases necessitate more careful analysis:

-
$\Omega_{T}^{R_{0}}$

**Fig 2:**
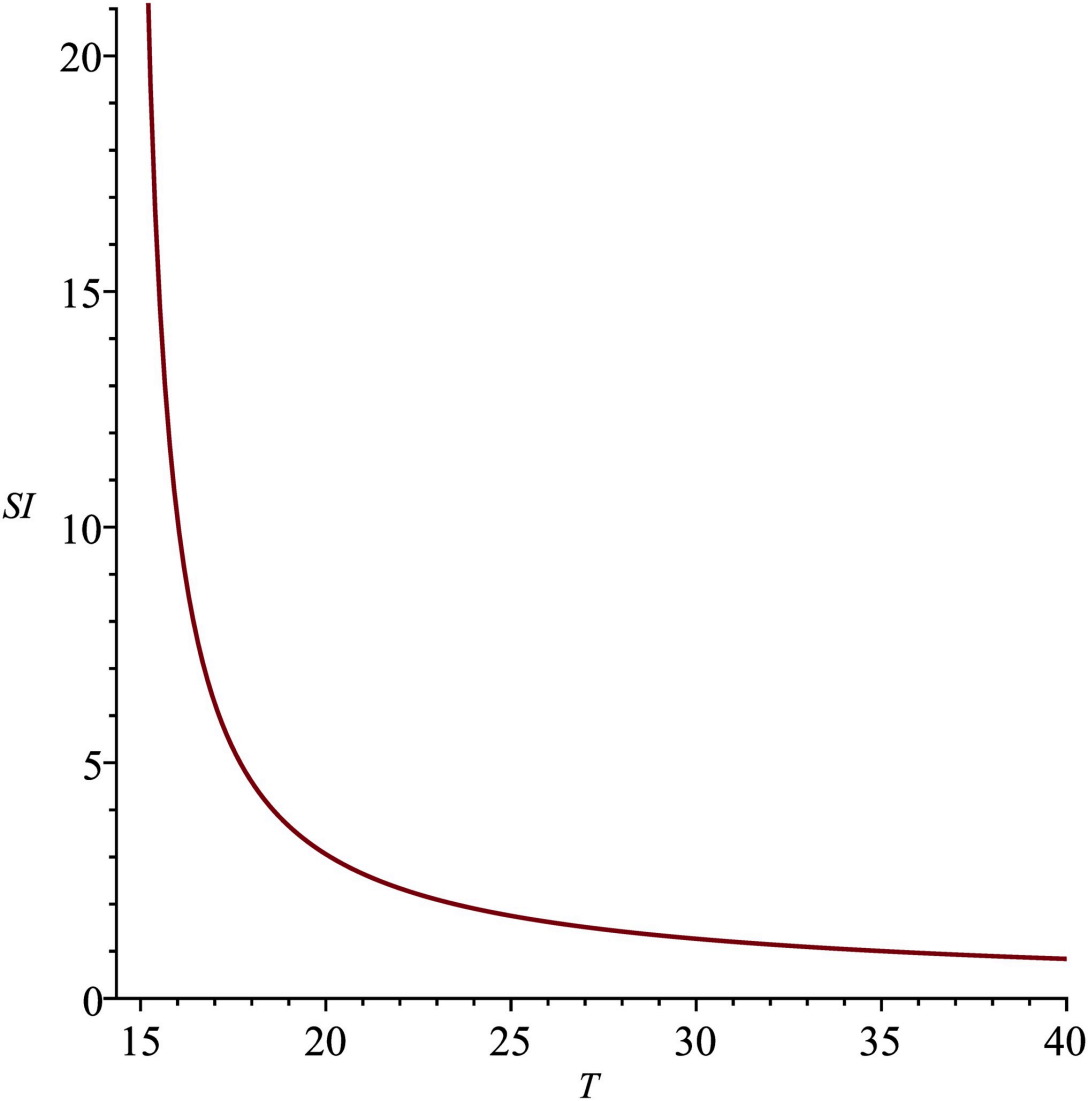
Sensitivity index $\Omega_{\text{T}}^{{\text{R}}_{0}}$.

  Temperature is directly related to the extrinsic latent period. For higher ambient temperatures, the extrinsic latent period decreases, being inversely proportional to *γ*. On the other hand, low temperatures below 14.5°*C* make it impossible for the parasite (*P.vivax*) to develop in the mosquito [32]. The relation between temperature T (°*C*), and the sensitivity index, $\Omega_{T}^{R_{0}}$ can be seen in Fig 2.
  For a temperature of 25°*C*, $\Omega_{T}^{R_{0}}=1.75$. Therefore, an increase of 10% in temperature (25°*C* to 27.5°*C*) implies an increase of 17.5% in *R*_0_.
  Environmental warming reduces the sensitivity of *R*_0_ to temperature ($\underset{T\to \infty }{\text{lim}}\frac{0.286\varepsilon T}{\left(0.286T-4.143)(0.286T-4.143+\varepsilon \right)}=0,\;\forall \varepsilon$). Obviously, *T* → ∞ is not a viable situation, it occurs due to simplifications in the modeling and estimation of coefficients that do not include mosquito and/or pathogen traits that are negatively affected by the increase in temperature. In this case, *T* → ∞ could be interpreted as an optimal *T* for disease proliferation.
  In colder environments, *R*_0_ is highly sensitive to temperature variations ($\underset{T\to \;14.5^{+}}{\text{lim}}\frac{0.286\varepsilon T}{\left(0.286T-4.143)(0.286T-4.143+\varepsilon \right)}=\infty ,\;\forall \varepsilon$), so that temperature variation can either make the extrinsic latent period compatible with the mosquito’s life expectancy, making it potentially infectious, or it can make these two periods incompatible, making it harmless. Below 23.5^*o*^*C*, temperature variations has a greater impact than the biting rate variations.
-
$\Omega_{\varepsilon }^{R_{0}}$

**Fig 3:**
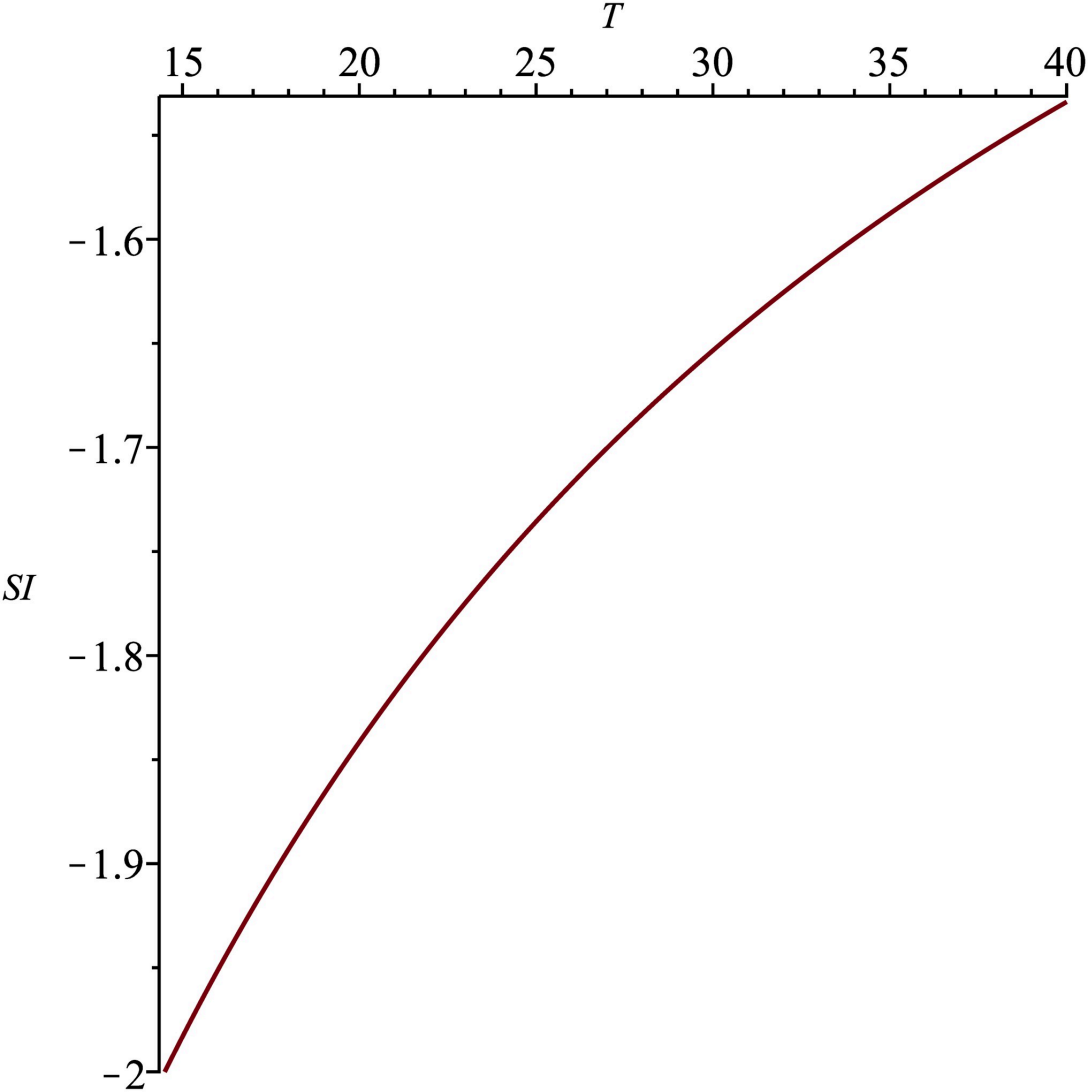
Sensitivity index $\Omega_{\varepsilon }^{{\text{R}}_{0}}$.

  The basic reproductive number, *R*_0_ is highly sensitive to variations in *ε* in colder environments. At 20°*C*, for example, an increase (decrease) of 10% in the recruitment rate to the adult stage, *ε*, induces a decrease (increase) of 18.4% in *R*_0_. However, at 30°*C*, an increase (decrease) of 10% in *ε* induces a decrease (increase) of 16.5% in *R*_0_. The relation between temperature and $\Omega_{\varepsilon }^{R_{0}}$ is shown in Fig 3. For any *ε* we have $\underset{T\to \infty }{\text{lim}}\Omega_{\varepsilon }^{R_{0}}=-1$ and $\underset{T\to 14.5}{\text{lim}}\Omega_{\varepsilon }^{R_{0}}=-2$.
-
$\Omega_{\mu }^{R_{0}}$

**Fig 4:**
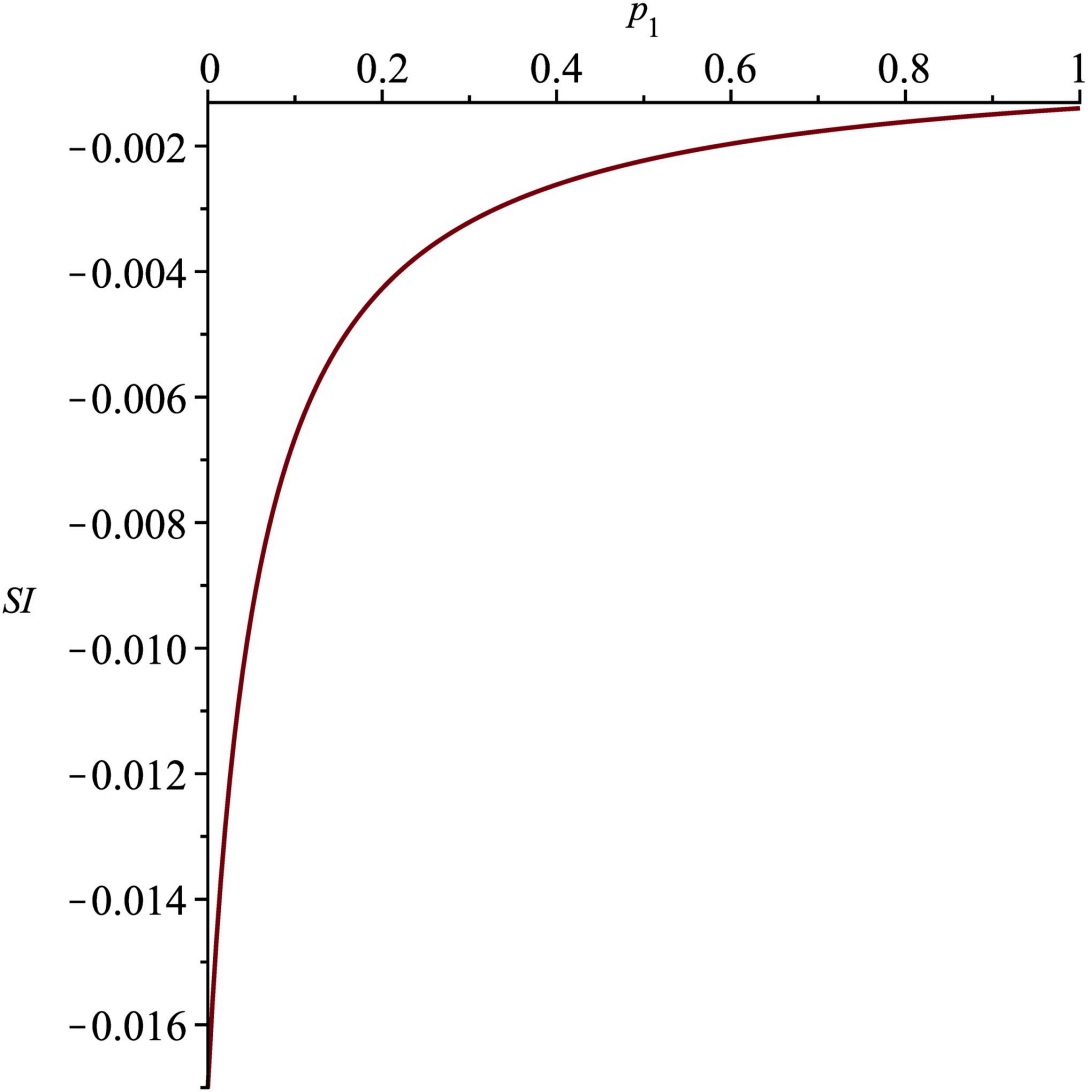
Sensitivity index $\Omega_{\mu }^{{\text{R}}_{0}}$ for p_1_ ∈ [0, 1], p_2_ = 1 − p_1_ and p_3_ = 0.

  The sensitivity index $\Omega_{\mu }^{R_{0}}$ depends on the proportion of people *p*_*κ*_ receiving a *κ*-treatment that induces a cure in 1/*ϕ*_*κ*_ months. The longer the cure time of the disease is, the more sensitive *R*_0_ is to *μ* variations, reaching $\Omega_{\mu }^{R_{0}}=-1$ in exceptional situations when the total absence of treatment leads to chronic malaria (*p*_1_ = *p*_2_ = 0, *p*_3_ = 1). In cases of rapid cure (*p*_1_ ↦ 1, *p*_2_, *p*_3_ ↦ 0), the sensitivity index $\Omega_{\mu }^{R_{0}}$ tends to zero; this observation means that if *μ* decreases by 10% (corresponding to an increase of 10% in life expectancy), the basic reproductive number may increase from 0 to 10%, depending on whether the cure is obtained over a shorter or longer period of time. Fig 4 illustrates this analysis.
-
$\Omega_{\phi_{\kappa }}^{R_{0}}$

**Fig 5:**
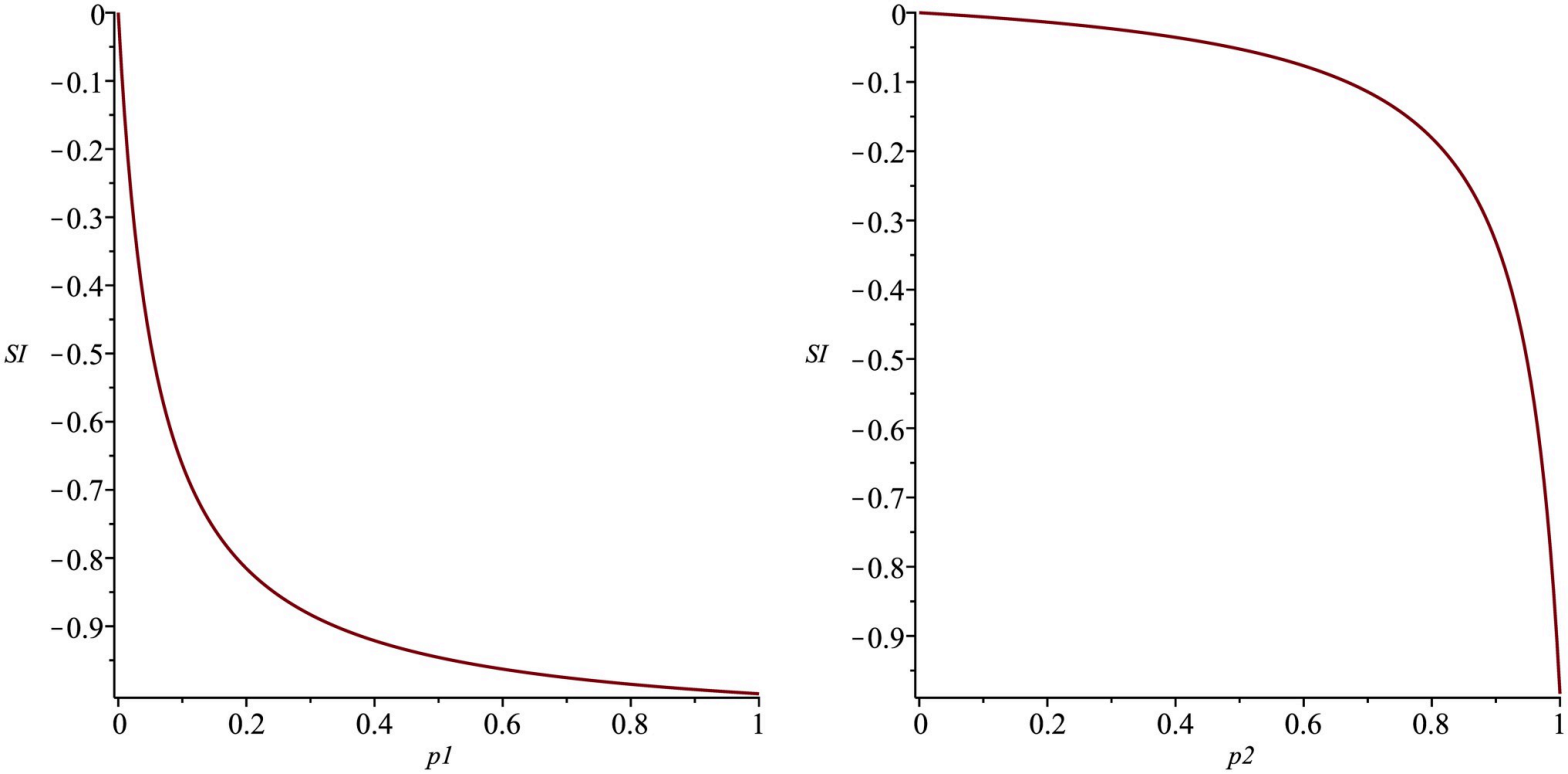
Sensitivity indexes $\Omega_{\phi_{1}}^{{\text{R}}_{0}}$ (a) and $\Omega_{\phi_{2}}^{R_{0}}$ (b), for p_1_ ∈ [0, 1], p_2_ = 1 − p_1_ and p_3_ = 0.

  *R*_0_ sensitivity to variations in *ϕ*_*κ*_ also depends on *p*_*κ*_. Note that the $\underset{p_{1},p_{2}\to 0;p_{3}\to 1}{\text{lim}}\Omega_{\phi_{\kappa }}^{R_{0}}=0$, ∀*κ*, and $\underset{p_{2},p_{3}\to 0;p_{1}\to 1}{\text{lim}}\Omega_{\phi_{\kappa }}^{R_{0}}$ is 0 for *κ* = 2, 3 or −0.99 for *κ* = 1. The last situation induces increased sensitivity of *R*_0_ to *ϕ*. In this $\Omega_{\phi_{\kappa }}^{R_{0}}\in ]-1,0]$. Fig 5 shows the sensitivity index of *R*_0_ to *ϕ*_1_ (a) and *ϕ*_2_ (b) for *p*_2_ = 1 − *p*_1_ and *p*_3_ = 0.
-
$\Omega_{p_{\kappa }}^{R_{0}}$

  The sensitivity of *R*_0_ to variations in *p*_*κ*_ follow the previous analysis for index $\Omega_{\phi_{\kappa }}^{R_{0}}$.

## 4. Results and discussion

This section deals with the results obtained from numerical simulations of the model (10) and (11), analyzing the influence of the realease of transgenic mosquitoes refractory to malaria into the ecosystem. The epidemiological behavior of malaria is shown in different scenarios, using the coefficients from Tables 2 and 4. The refractoriness of transgenic mosquitoes is assumed to be 80%, independent of zygosity or genetic technique (Mendelian or MCR inheritance); thus, the probability of infection for transgenic mosquitoes is *ζ*_2_ = *ζ*_3_ = 0.2*ζ*_1_. This assumption is in line with research carried out by Ito et al. [16].

**Table 4:** Genotypic frequencies for both Mendelian and MCR inheritance.

Frequency	*a*_11_	*a*_12_	*a*_13_	*a*_22_	*a*_23_	*a*_33_
Mendelian	1.0	0.5	0.0	0.25	0.0	0.0
MCR	1.0	$\frac{0.5}{w_{12}}$	0.0	$\frac{0.25}{w_{22}}$	0.0	0.0
Frequency	*b*_11_	*b*_12_	*b*_13_	*b*_22_	*b*_23_	*b*_33_
Mendelian	0.0	0.5	1.0	0.5	0.5	0.0
MCR	0.0	$\frac{0.5(1-c)(1-hs)}{w_{12}}$	$\frac{1.0(1-c)(1-hs)}{w_{13}}$	$\frac{0.5(1-c)(1-hs)}{w_{22}}$	$\frac{0.5(1-c)(1-hs)}{w_{23}}$	0.0
Frequency	*c*_11_	*c*_12_	*c*_13_	*c*_22_	*c*_23_	*c*_33_
Mendelian	0	0.0	0.0	0.25	0.5	1.0
MCR	0	$\frac{0.5c(1-s)}{w_{12}}$	$\frac{1.0c(1-s)}{w_{13}}$	$\frac{\left(0.25+0.5c)(1-s\right)}{w_{22}}$	$\frac{\left(0.5+0.5c)(1-s\right)}{w_{23}}$	1.0

Table 4 shows the genotypic frequencies evaluated for classical Mendelian genetics and the MCR technique. Assuming to Mendelian inheritance, we have the following results:

- mating *u*_1_ × *u*_1_ generating wild mosquitos with genotypic frequency *a*_11_ = 1.0;
- mating *u*_1_ × *u*_2_ generating wild mosquitos with genotypic frequency *a*_12_ = 0.5 and heterozygous mosquitoes with genotypic frequency *b*_12_ = 0.5;
- mating *u*_2_ × *u*_2_ generating wild mosquitos with genotypic frequency *a*_22_ = 0.25, heterozygous mosquitoes with genotypic frequency *b*_22_ = 0.5 and homozygous transgenic mosquitoes with genotypic frequency *c*_22_ = 0.25;
- mating *u*_1_ × *u*_3_ generating heterozygous mosquitoes with genotypic frequency *b*_13_ = 1.0;
- mating *u*_2_ × *u*_3_ generating heterozygous mosquitoes with genotypic frequency *b*_23_ = 0.5 and homozygous transgenic mosquitoes with genotypic frequency *c*_23_ = 0.5;
- mating *u*_3_ × *u*_3_ generating homozygous transgenic mosquitoes with genotypic frequency *c*_33_ = 1.0;

According to MCR inheritance, the conversion of heterozygous mosquitoes to homozygous transgenic mosquitoes occurs with a success rate of *c*; so, the resistance to this conversion is 1 − *c*. *w*_*ij*_ is the mean population fitness associated with population obtained from mating *U*_*i*_ × *U*_*j*_ and the respective selection coefficients for genotypes (*w*, *w*), (*g*, *g*) and (*g*, *w*) are 1, 1 − *s* and 1 − *hs*, where *s* is the selection coefficient and *h* is the degree of dominance of fitness cost.

The efficiency of converting heterozygous into homozygous transgenic mosquitoes for *A. stephensi* was 98% when transgenic males mate wild females and 14% when wild males mate transgenic females [22, 23].

In the following simulations, we consider the worst-case scenarios of *c* = 0.14 (MCR); note that *c* = 0 implies Mendelian inheritance.

### 4.1 Simulations of mosquitoes population using Mendelian and MCR inheritance

Each set of figures in this section compares model simulations (1) for four situations: (1) the epidemiological dynamics of wild mosquitoes only, (2) the epidemiological dynamics of wild and transgenic mosquitoes using classical Mendelian genetics, (3) the epidemiological dynamics of wild and transgenic mosquitoes using MCR inheritance without fitness cost and (4) the epidemiological dynamics of wild and transgenic mosquitoes using MCR inheritance and fitness cost. The initial conditions were assumed to have a proportion *m* = 11.57 between mosquitoes and humans, where the human population has constant size, H*, and the mosquito population stabilizes at U* given by Eq (3); thus $\left(H_{S_{0}},H_{E_{0}},H_{I_{0}}\right)=\left(9990,0,10\right)$ for humans and $\left(U_{1S_{0}},U_{2S_{0}},U_{3S_{0}}\right)=\left(21000,0,0\right)$ for situation (1), $\left(U_{1S_{0}},U_{2S_{0}},U_{3S_{0}}\right)=\left(10000,11000,10^{-5}\right)$ for situation (2) and $\left(U_{1S_{0}},U_{2S_{0}},U_{3S_{0}}\right)=\left(10000,11000-11000c,10^{-5}+11000c\right)$ for situations (3) and (4); the other populations were initially assumed to be zero. In addition, the parameter values from Tables 2 and 4 were used.

**Fig 6:**
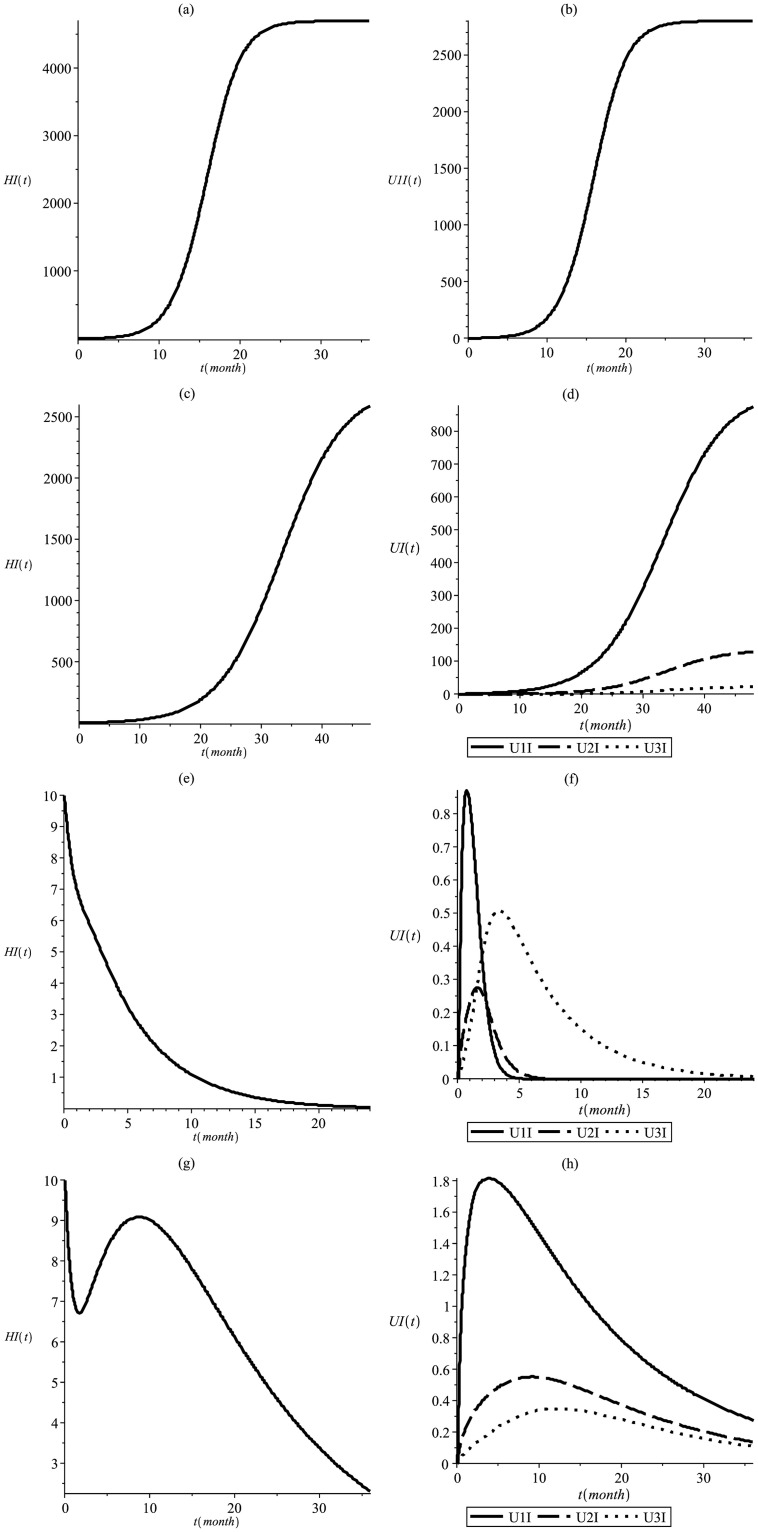
This simulation shows the infectious dynamics at T = 25°C and p_1_ = 0.3, p_2_ = 0.4, p_3_ = 0.3. Infectious human (a) and wild mosquitoes (b) in the absense of transgenic, resulting in *R*_0_ = 2.43; infectious human (c) and mosquitoes (d) considering Mendelian inheritance and resulting in *R*_*Mend*_ = 1.54; infectious human (e) and mosquitoes (f) considering MCR inheritance and without fitness cost resulting in *R*_*MCR*_ = 0.48; infectious human (g) and mosquitoes (h) considering MCR inheritance and fitness cost resulting in *R*_*MCR*_ = 0.83.

Fig 6 shows the infectious dynamics at a temperature of *T* = 25°*C* and disease recovery due to treatment in proportions of *p*_1_ = 0.3, *p*_2_ = 0.4 e *p*_3_ = 0.3, which means that 30% of the infected people sucessfully eliminated the protozoan from their bloodstream in 15 days, 40% of infected people only succeeded in eliminating the protozoan after 2 years of precarious treatment and 30% of the infected people were unable to eliminate the protozoan (it is common to find asymptomatic people in this situation). This configuration leads to *R*_0_ = 2.43, *R*_*Mend*_ = 1.54 and *R*_*MCR*_ = 0.48 with no fitness cost (*s* = 0). When *s* = 0.35 and *h* = 0.2 the mosquito population stabilizes in a proportion $\left(u_{1}^{*},u_{2}^{*},u_{3}^{*}\right)=\left(0.18,0.45,0.37\right)$, implying *R*_*MCR*_ = 0.83. This result suggests that the epidemic reaches a smaller number of people when transgenic mosquitoes are released next to a mosquito breeding site. The use of transgenic mosquitoes with Mendelian inheritance reduced the number of infected people by 42% after 4 years. Transgenic mosquitoes with MCR inheritance were more successful, as expected, being able to contain the contagion in approximately 2 years without fitness cost and 3 years with fitness cost.

**Fig 7:**
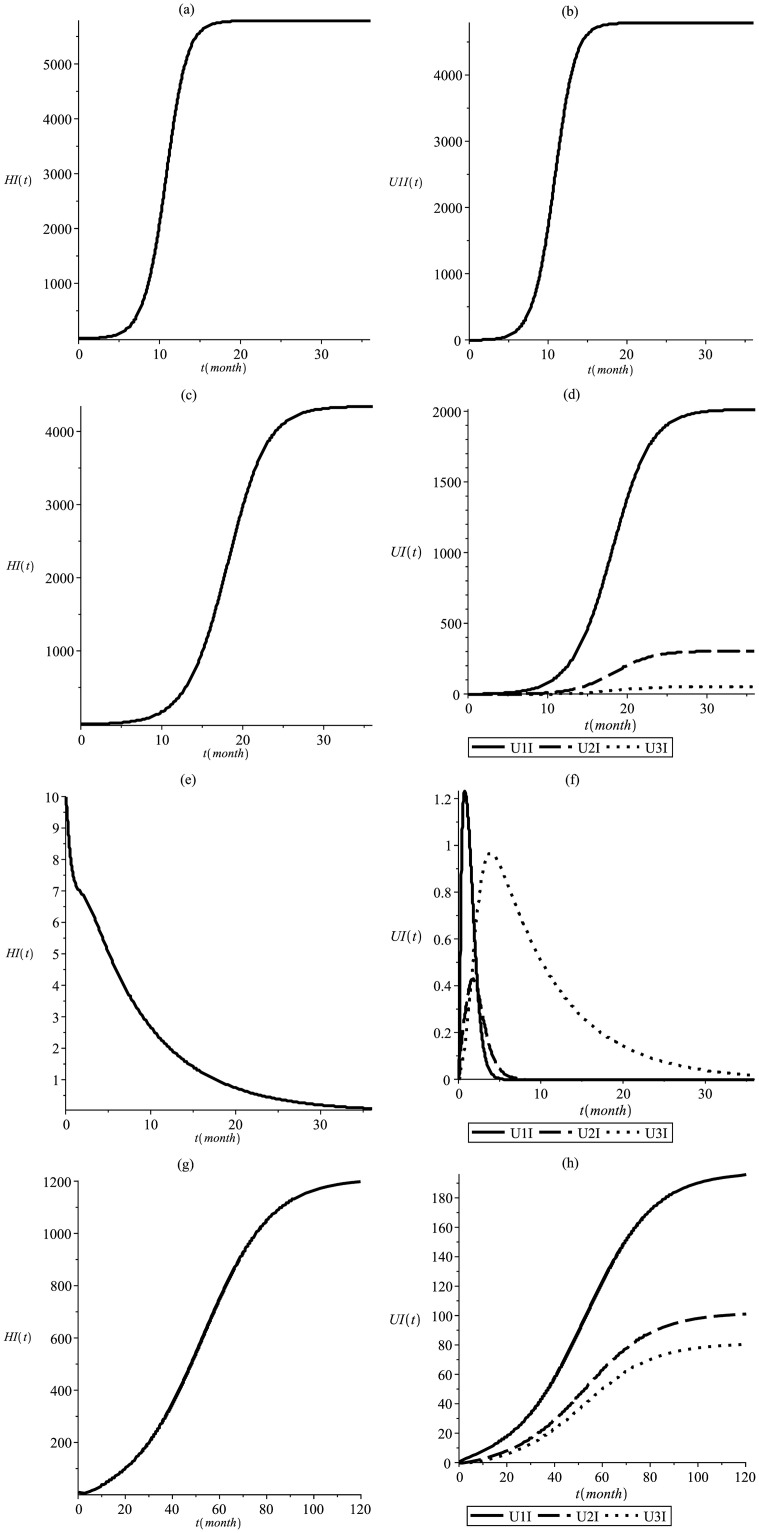
This simulation shows the infectious dynamics at T = 32°C and p_1_ = 0.3, p_2_ = 0.4, p_3_ = 0.3. Infectious human (a) and wild mosquitoes (b) in the absense of transgenic, resulting in *R*_0_ = 3.44; infectious human (c) and mosquitoes (d) considering Mendelian inheritance and resulting in *R*_*Mend*_ = 2.19; infectious human (e) and mosquitoes (f) considering MCR inheritance and without fitness cost resulting in *R*_*MCR*_ = 0.69; infectious human (g) and mosquitoes (h) considering MCR inheritance and fitness cost resulting in *R*_*MCR*_ = 1.18.

In Fig 7, the same four situations are considered, as well as the initial conditions. In this case the environmental temperature increased to *T* = 32°*C* and recovery due to treatment was maintained in the proportions of *p*_1_ = 0.3, *p*_2_ = 0.4 e *p*_3_ = 0.3. This configuration leads to *R*_0_ = 3.44, *R*_*Mend*_ = 2.19, *R*_*MCR*_ = 0.69 with no fitness cost and *R*_*MCR*_ = 1.18 for *s* = 0.35 and *h* = 0.2. This values for the reproductive number evidence the influence of temperature on the epidemiological process. As shown in Section 4.1, threshold *R*_0_ is highly sensitive to temperature, which is directly related to the extrinsic latent period, causing it to be reduced and rendering the mosquito a younger infectious agent. Despite efforts to genetically manipulate mosquitoes, the epidemiological numbers remain troubling high, although the delay in the occurrence of the epidemic is significant due to the release of transgenic mosquitoes. In the only situation in which the epidemic was prevented (Fig 7e and 7f) transgenic mosquitoes without fitness cost were released.

**Fig 8:**
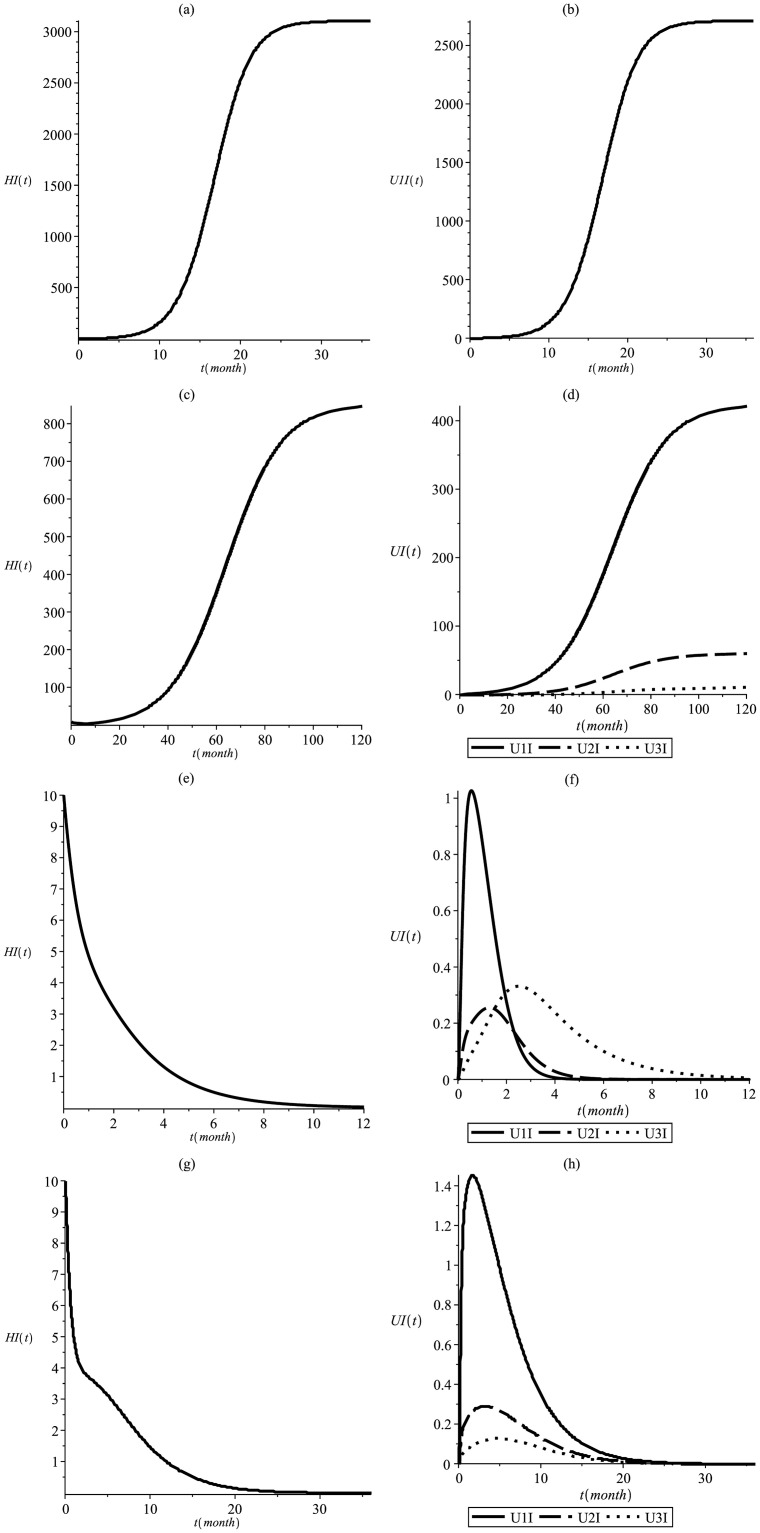
This simulation shows the infectious dynamics at T = 32°C and p_1_ = 0.6, p_2_ = 0.3, p_3_ = 0.1. Infectious human (a) and wild mosquitoes (b) in the absense of transgenic, resulting in *R*_0_ = 1.8; infectious human (c) and mosquitoes (d) considering Mendelian inheritance and resulting in *R*_*Mend*_ = 1.14; infectious human (e) and mosquitoes (f) considering MCR inheritance and without fitness cost resulting in *R*_*MCR*_ = 0.36; infectious human (g) and mosquitoes (h) considering MCR inheritance and fitness cost resulting in *R*_*MCR*_ = 0.62.

Fig 8 shows that improvements in health care are also important tools for controlling epidemiological processes. Maintaining a temperature of 32°*C* and adopting *p*_1_ = 0.6, *p*_2_ = 0.3 and *p*_3_ = 0.1, the levels of infectious humans and mosquitoes are quickly controlled, reaching *R*_0_ = 1.8, *R*_*Mend*_ = 1.14, and *R*_*MCR*_ = 0.36 when no fitness cost is associated and *R*_*MCR*_ = 0.62 for *s* = 0.35 and *h* = 0.2. In this situation transgenic mosquitoes with Mendelian inheritance reduced the number of infected people by 72% and restrained the epidemic, which only reached its peak after 10 years, ensuring time for other actions. The MCR transgenic mosquitoes were very successful, even considering fitness cost, eliminating the disease in one year for the first case (without fitness cost) and three years for the second one (with fitness cost).

## 5. Conclusions

This paper presents a mathematical model to explore malaria evolution in the presence of transgenic mosquitoes, extending the approach previously introduced by Wyse et al. [27] and coupling the mosquito dynamics described in the recent paper by Wyse et al. [25]. The mosquito offspring can follow classical Mendelian genetics or be obtained by MCR, which converts heterozygous individuals to homozygous individuals at an immature stage.

To study disease dynamics, two equilibrium points were identified: endemic equilibrium and free-disease equilibrium. A qualitative study of these equilibrium points led us to obtain a stability condition that characterizes the basic reproductive rate or, in the presence of transgenic mosquitoes, the effective reproductive rate.

The main purpose was to evaluate the impact of transgenic mosquitoes in reinforcing decreased malaria activity together with other factors included in the model.

The extrinsic latent period plays a critical role because it is highly sensitive to temperature variation. It is evident from the model that an elevation of the temperature is a critical factor contributing to epidemic risk, althougt temperature can affect numerous important entomological parameters which are not considered in the proposed model, not just the extrinsic latent period. Mordecai et al. [33] consider some mosquito and pathogen traits are temperature sensitive (survival, development, reproduction), then there is an interval where the temperature is adequate to disease transmission. It means that high temperature reduce the extrinsic latent period but also reduce mosquito life span and other mosquito traits. The result of the sensitivity of *R*_0_ to the temperature is in according with the studies conduced by Mordecai et al. [33] and Paaijmansa et al. [34] since a lower limit until an optimal temperature. Negative effects from an upper limit for the temperature are not been considered, so that the model considers only extrinsic latent period to be temperature-dependent.

Thus, decision makers’ attitudes must extend beyond exclusively fighting disease. In addition, the model draws attention to the consequences of global climatic change on human health, which could take malaria beyond the tropics and worsen the situation in areas that are already at risk. Certainly, transgenic mosquitoes are an alternative to reduce the disease, but, for highly transmissible a small vector population of wild mosquitoes is sufficient to introduce the risk of epidemic conditions.

For offspring following Mendelian genetics, it is impossible to reach an equilibrium where the mosquito population is completely transgenic unless the initial condition for the model assumes that all the mosquitoes are homozygous transgenic organisms [35], which is practically impossible. The offspring obtained by the MCR technique are more successful, since any *c* > 0 causes the wild mosquito population to be suppressed by homozygous transgenics within a particular time frame if no fitness cost is associated to transgenic variety or the fitness cost is compensated with other factors related to disease control; in this case, adequate treatment has been shown to be effective in conjunction with transgenic mosquitoes.

Prophylaxes and drugs exist for many diseases, including malaria, and are important for the reduction of disease incidences. Rapid diagnoses and medical treatment compliance as recommended by health authorities are determinant factors in disease control. The sole use of transgenic mosquitoes cannot be enough for disease eradication. Under certain circumstances, it is necessary to combine appropriate measures and improve the genetic engineering of transgenic mosquitoes, perhaps guaranteeing high refractoriness to protozoans.
